# Metabolic reprogramming by pseudorabies virus: nucleotide metabolism as a central vulnerability for viral replication and pathogenesis

**DOI:** 10.3389/fmicb.2025.1683365

**Published:** 2025-11-28

**Authors:** Xiaoyong Chen, Qingsen Wang, Xi Chen

**Affiliations:** 1 Xingzhi College, Zhejiang Normal University, Jinhua, China; 2 Institute of Animal Husbandry and Veterinary Medicine, Fujian Academy of Agriculture Sciences, Fuzhou, China

**Keywords:** LC–MS, metabolites, pseudorabies virus, nucleotide metabolism, PK-15 cells

## Abstract

Pseudorabies virus (PRV), a neurotropic alphaherpesvirus, causes severe neurological and reproductive disorders in swine, posing substantial threats to the global swine industry and emerging as a zoonotic concern. Viral metabolic reprogramming is a conserved strategy to support replication, yet the metabolic landscape of PRV infection remains incompletely defined. Here, we employed ultrahigh-performance liquid chromatography-mass spectrometry (UPLC–MS)-based metabolomics combined with multivariate statistical analysis to systematically profile metabolic changes in PRV-infected porcine kidney PK-15 cells. Unsupervised principal component analysis (PCA) and supervised orthogonal partial least squares-discriminant analysis (OPLS-DA) revealed a striking separation of metabolic phenotypes between PRV-infected (48 hpi) and control (0 hpi) cells, with the first principal component accounting for >73% of total variance, confirming significant metabolic reprogramming upon infection. We identified 1,634 and 925 differential metabolites in ESI+ and ESI− modes, respectively, with hierarchical cluster analysis revealing distinct signatures: amino acids and heterocyclic compounds were predominantly upregulated, while glycerophospholipids (GPs) were markedly downregulated. KEGG pathway enrichment analysis highlighted key perturbed networks underlying PRV pathogenesis: glycerophospholipid metabolism was targeted to modulate membrane dynamics for viral egress and dampen innate immune signaling; aminoacyl-tRNA and nucleotide sugar metabolism were enhanced to support viral protein synthesis and glycosylation. Notably, nucleotide metabolism was profoundly upregulated, with increased levels of adenosine, guanosine, adenine, and xanthosine. Transcriptomic validation (GSE8676 dataset) and qRT-PCR confirmed time-dependent upregulation of critical purine biosynthesis genes, including IMPDH, GMPS, ADSS2, GART, and ATIC, peaking at 48 hpi. These findings demonstrate that PRV orchestrates multifaceted metabolic takeover, with nucleotide metabolism emerging as a key vulnerability. Targeting this pathway may offer novel strategies to disrupt PRV replication, providing insights into viral-host metabolic crosstalk and antiviral development.

## Introduction

Pseudorabies virus (PRV) is a member of the *Alphaherpesvirinae* family responsible for pseudorabies (PR), which has severely harmed the global swine industry (Chen et al., 2021). It infects a wide range of hosts and induces cytopathic alterations in infected cells. It is interesting to note that it’s an important model virus for investigating the biological traits of herpesviruses. Pigs, the only natural host, exhibit clinical manifestations ranging from non-specific symptoms to fatal infection (Tan et al., 2021). However, young piglets and susceptible animals from non-native host species usually die from PRV infection due to deadly neurotoxicity (Sun et al., 2019). In recent years, attenuated vaccines with deleted virulence genes and serological diagnostic tests have been used worldwide to prevent PRV infection in pigs (Yu et al., 2017). However, due to ongoing immunological pressure, novel strains of PRV variants are still discovered in clinical settings, creating new challenges for PR prevention and treatment. Moreover, it has been demonstrated that PRV infection can spread to people and result in disorders of the central nervous system (CNS), such as endophthalmitis and fatal encephalitis (Yu et al., 2017).

Metabolomics aims to provide as much information as possible about the metabolic responses of biological systems to genetic changes or pathogenic stressors (Beale et al., 2018). It is a supplementary method to genomics, which focuses on the genome and gene expression, and proteomics, which investigates how various stimuli impact cellular proteins The host genetic and environmental factors influence the metabolic profile (Johnson et al., 2016). Because of this, metabolomics has great promise for application in critical care medicine, where patients are complicated and understanding the interactions between illness, medication effects, and host characteristics is essential to providing improved care. Metabolomics has the potential to improve diagnosis and treatment monitoring and provide a deeper understanding of disorders that may be possible with a single biomarker or small range of biomarkers (Pei et al., 2017; Bauermeister et al., 2022). Therefore, it might be easier to understand the characteristics of the infections and how diseases develop by studying the metabolic pathways.

A growing body of evidence has shown that multiple viruses are capable of manipulating host components and signal pathways to boost viral replication by reprogramming host metabolism. For instance, Newcastle disease virus (NDV) regulates the metabolism of host cells by degrading sirtuin 3 (SIRT3) via PTEN-induced kinase 1 (PINK1)- Parkin (PRKN)-dependent mitophagy (Gong et al., 2022). By promoting the uptake of glucose and glutamine by cells, porcine reproductive and respiratory syndrome virus (PRRSV) infection supplies the energy and macromolecules required for self-replication. In addition, PRRSV effective replication in cells depends on glycolysis and the tricarboxylic acid (TCA) cycle (Pang et al., 2023). Porcine epidemic diarrhea virus (PEDV) infection suppressed transcription of lipid metabolism-related genes, and treatment of the infected enteroids by palmitic acid led to pronouncedly reduction of PEDV replication. These findings suggest that PEDV may take advantage of the host lipid metabolism for its own replication (Mao et al., 2022). In our recent study, we found that PRV infection disrupted the succinylation of cellular enzymes that are critical to metabolic processes (Chen et al., 2023). However, despite these advances, the role of nucleotide metabolism—a pathway fundamental to viral DNA replication—remains incompletely characterized in the context of PRV infection. Nucleotides are the building blocks of viral genomes, and their *de novo* synthesis or salvage by host cells is often a rate-limiting step for viral replication. Herpesviruses, in particular, are known to upregulate nucleotide metabolic pathways to support their replicative cycle, yet the specific mechanisms by which PRV modulates these pathways, and their significance for viral fitness, remain to be defined.

In this study, we employed ultrahigh-performance liquid chromatography-mass spectrometry (UPLC–MS)-based metabolomics to systematically profile the metabolic landscape of PRV-infected porcine kidney PK-15 cells, a well-established *in vitro* model for PRV research. By integrating multivariate statistical analysis and pathway enrichment, we aimed to identify key metabolic pathways perturbed by PRV infection. We further focused on characterizing changes in nucleotide metabolism and validated the expression of critical enzymes involved in purine biosynthesis using quantitative real-time PCR (qRT-PCR). Our findings reveal a profound reprogramming of host metabolism upon PRV infection, with a striking upregulation of nucleotide metabolism, highlighting this pathway as a potential target for antiviral strategies.

## Materials and methods

### Viruses and cells

Porcine kidney PK-15 cells (CCL-33) were purchased from the American Type Culture Collection (ATCC, USA). The cells were cultured in Dulbecco’s Modified Eagle Medium (DMEM, Bioland) supplemented with 10% (v/v) heat-inactivated fetal bovine serum (FBS, KX-A1220, Inner Mongolia Wanrui Biotechnology Co., Ltd.) and 1% (v/v) penicillin-streptomycin solution (Biolight). Cells were grown in tissue culture flasks (CellPro Biotechnology) and maintained in a humidified incubator at 37°C with 5% CO_2_.

PRV RA strain was preserved in our laboratory (Chen et al., 2023). PK-15 cells grown at 90% confluence were challenged with viruses at a multiplicity of infection (MOI) of 1. Then they were incubated at 37 °C for 1 h. Next, the unattached viruses were washed away using phosphate buffer saline (Wuhan SUNNCELL Biotechnology Co., Ltd). Then cells were maintained in DMEM containing 2% FBS at 37 °C before harvest.

### Metabolomics

The PK-15 cells were infected and collected from two groups at 0 h post infection (hpi) and 48 hpi, namely the 0 h group and the 48 h group, with three replicates in each group and each replicate containing more than 1 × 10^7^ cells. After discarding the supernatant, the cells were washed three times with PBS. Then, they were collected using cell scraper (BaiDi Biotechnology Co., Ltd.) and placed in 1.5 mL centrifuge tubes(3041100, Zhejiang Saining Biotechnology Co., Ltd.). These cell-containing tubes were immediately placed in liquid nitrogen and then shipped to Metware (Wuhan, China) under dry ice.

Following thawing on ice, the samples were combined with 1 mL of a pre-chilled extraction solution of methanol, acetonitrile, and water in a 2:2:1 volume ratio. The resulting homogenate was then subjected to ultrasonication for 30 min and subsequently centrifuged at 14,000 g for 20 min at 4 °C to pellet insoluble debris. The clarified supernatant was collected, dried under vacuum centrifugation, and the resulting metabolite pellet was reconstituted in 100 μL of a 50% (v/v) acetonitrile aqueous solution prior to LC–MS analysis.

All samples were analyzed using an LC–MS system following the manufacturer’s recommended protocols. For UPLC analysis, a Waters ACQUITY UPLC HSS T3 C18 column (1.8 μm particle size, 2.1 mm × 100 mm) was employed, with the column temperature maintained at 40 °C, a flow rate of 0.4 mL/min, and an injection volume of 2 μL. The mobile phase system comprised two components: phase A (water supplemented with 0.1% formic acid) and phase B (acetonitrile supplemented with 0.1% formic acid). The gradient elution program was conducted as follows: initial conditions at 0 min consisted of 5% mobile phase B, followed by a linear gradient increase to 90% mobile phase B over 11 min. This 90% mobile phase B condition was held for 1 min, after which the system was rapidly reverted to 5% mobile phase B within 0.1 min and maintained at this composition for an additional 1.9 min to re-equilibrate the column.

### Bioinformatic analysis

Using ProteoWizard software, the original data file acquired by LC–MS was transformed into mzML format. The XCMS program was used to carry out peak extraction, peak alignment, and retention time correction. The peak area was adjusted using the “SVR” approach. In each sample group, the peaks with a detection rate of less than 50% were eliminated. After that, the laboratory’s in-house created database, integrated public database, AI database, and metDNA searches were used to find information about metabolic identification.

### Principal component analysis (PCA)

Using R’s statistics function, unsupervised principal component analysis (PCA) was carried out.^1^ Prior to unsupervised PCA, the data were scaled using the unit variance.

### Hierarchical cluster analysis (HCA) and Pearson correlation coefficients (PCC)

While PCC between samples were calculated using R’s cor function, the HCA findings of the samples and metabolites were displayed as heatmaps with dendrograms. Using the R package Complex Heatmap, both HCA and PCC were performed. A color spectrum represents the adjusted signal intensities of metabolites (unit variance scaling) for HCA.

### Differential metabolites selected

Differential metabolites for the two-group analysis were found using VIP (VIP > 1) and *p*-value (*p* < 0.05, Student’s *t*-test). Using the R package MetaboAnalystR, the OPLS-DA result—which also included score and permutation plots—was extracted to get VIP values. Before OPLS-DA, the data underwent mean centering and log transform (log2). A permutation test with 200 permutations was carried out to prevent overfitting.

### KEGG annotation and enrichment analysis

The KEGG Compound database^2^ was used to annotate the identified metabolites. The KEGG Pathway database^3^ was then mapped to the annotated metabolites. For a given set of metabolites, the *p*-value of a hypergeometric test identifies significantly enriched pathways.

### Quantitative real-time PCR

Total RNA was extracted from PK-15 cells using the RNA Isolation Kit (Jinan, Shandong Sparkjade Biotechnology Co., Ltd.) following the instructions. For reverse transcription, complementary DNA (cDNA) was synthesized using ToloScript All-in-one RT EasyMix for qPCR (Shanghai, Tolo Biotech Co., Ltd) according to the instructions. The quantitative real-time PCR was performed using Universal SYBR qPCR Master Mix (Q411-W, Nanjing, ATG Biotechnology Co., Ltd.) together with the primers listed in Table 1. Glyceraldehyde 3-phosphate dehydrogenase (GAPDH) serves as reference gene to normalize target gene expression (Bai et al., 2020). The RNA levels of genes were calculated through LightCycler^®^ 96 system (Roche, Switzerland).

**Table 1:** Primers used in this study.

Name	Accession	Sequences (5′-3′)	Product size (bp)
GAPDH-F	NM_001206359.1	TACACTGAGGACCAGGTTGTG	101
GAPDH-R		TTGACGAAGTGGTCGTTGAG	
IMPDH-F	XM_021078710.1	GGGCTCTTTCATGGCGGA	129
IMPDH-R		CCTGGGAGAATCAGGAAGTCG	
GMPS-F	XM_021069704.1	TCCAGACCTACCAAGAGAACCA	102
GMPS-R		TCCTTAAGGTCTCCTCCAGCA	
ADSS2-F	NM_001097508.1	TGAGGCTTTACATGGGCCAC	133
ADSS2-R		CAAGCCAGTGCAAACACCTC	
GART-F	XM_021070945.1	ACATTGGCTGTCGTGCCATA	81
GART-R		CAGCTGCAATGTCTACCCCA	
ATIC-F	NM_001130736.1	TTGGTGGGGTAACCTTGCTG	113
ATIC-R		GTTGTCAGAGCCCTGCATCT	

### Statistical analysis

Three separate experiments are represented by the mean ± standard deviation (S.D.) for the data. Analysis of variance (ANOVA) was used in the statistical analysis, and the GraphPad Prism 5 program (GraphPad Software, USA) was used. Statistical significance was indicated by a *p* value of less than 0.05.

## Results

### Principal component analysis of PRV-infected PK-15 cells

**Figure 1:**
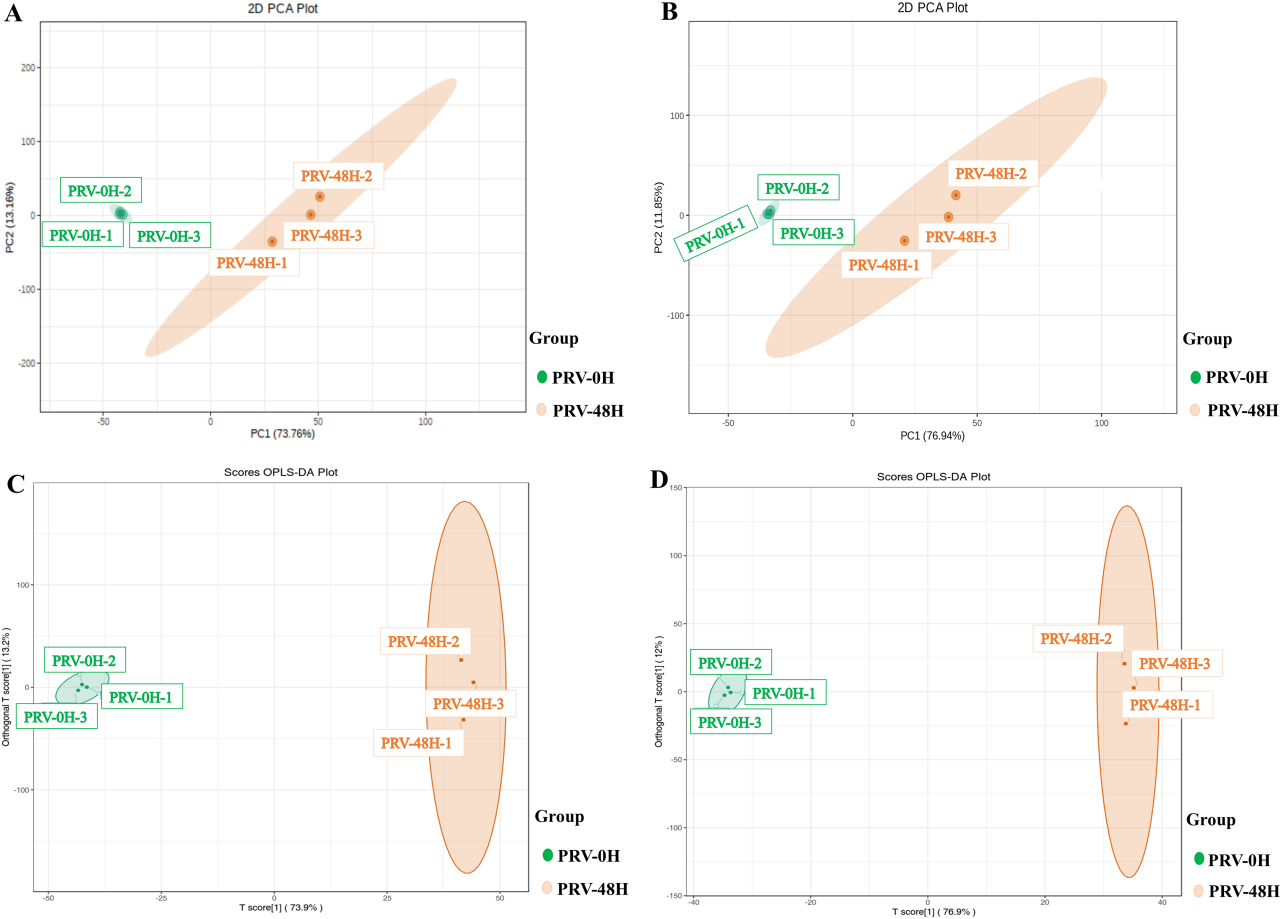
Multivariate statistical analysis of the metabolic profiles. PCA score plots of metabolic profiles from PRV-infected PK-15 cells (48 h post-infection, PRV-48H) and mock-infected controls (0 hpi, PRV-0H) in positive (ESI+, **A**) and negative (ESI−, **B**) ionization modes. Each point represents an independent biological replicate (*n* = 3). The percentage of variance explained by each principal component (PC) is indicated on the axes. **(C,D)** Orthogonal projections to latent structures-discriminant analysis (OPLS-DA) score plots derived from the same datasets in ESI+ **(C)** and ESI− **(D)** modes. The model quality parameters are displayed.

To evaluate the global impact of PRV infection on the metabolic profile of PK-15 cells, we performed unsupervised principal component analysis (PCA) and supervised orthogonal partial least squares-discriminant analysis (OPLS-DA) under both ESI+ and ESI− modes. PCA score plots revealed a clear separation between the 0 hpi control group and 48 hpi PRV-infected group in both ionization modes (Figures 1A,B). Specifically, the first principal component (PC1) accounted for 73.8% of the total variance in ESI+ mode and 76.9% in ESI− mode, indicating that metabolic differences induced by PRV infection were the primary driver of sample separation.

To further refine the discrimination, OPLS-DA was applied, which yielded robust models with high explanatory power (R^2^X = 0.87 in ESI+; R^2^X = 0.89 in ESI−) and predictive ability (Q^2^ = 0.99 in ESI+; Q^2^ = 0.99 in ESI−) (Figures 1C,D). A permutation test with 200 iterations confirmed no overfitting (*p* < 0.01), validating the reliability of the models. Collectively, these results demonstrated that PRV infection significantly altered the metabolic phenotype of PK-15 cells, confirming the successful establishment of the infection model for subsequent analyses.

### Comprehensive screening of differential metabolites in PRV infection

**Figure 2:**
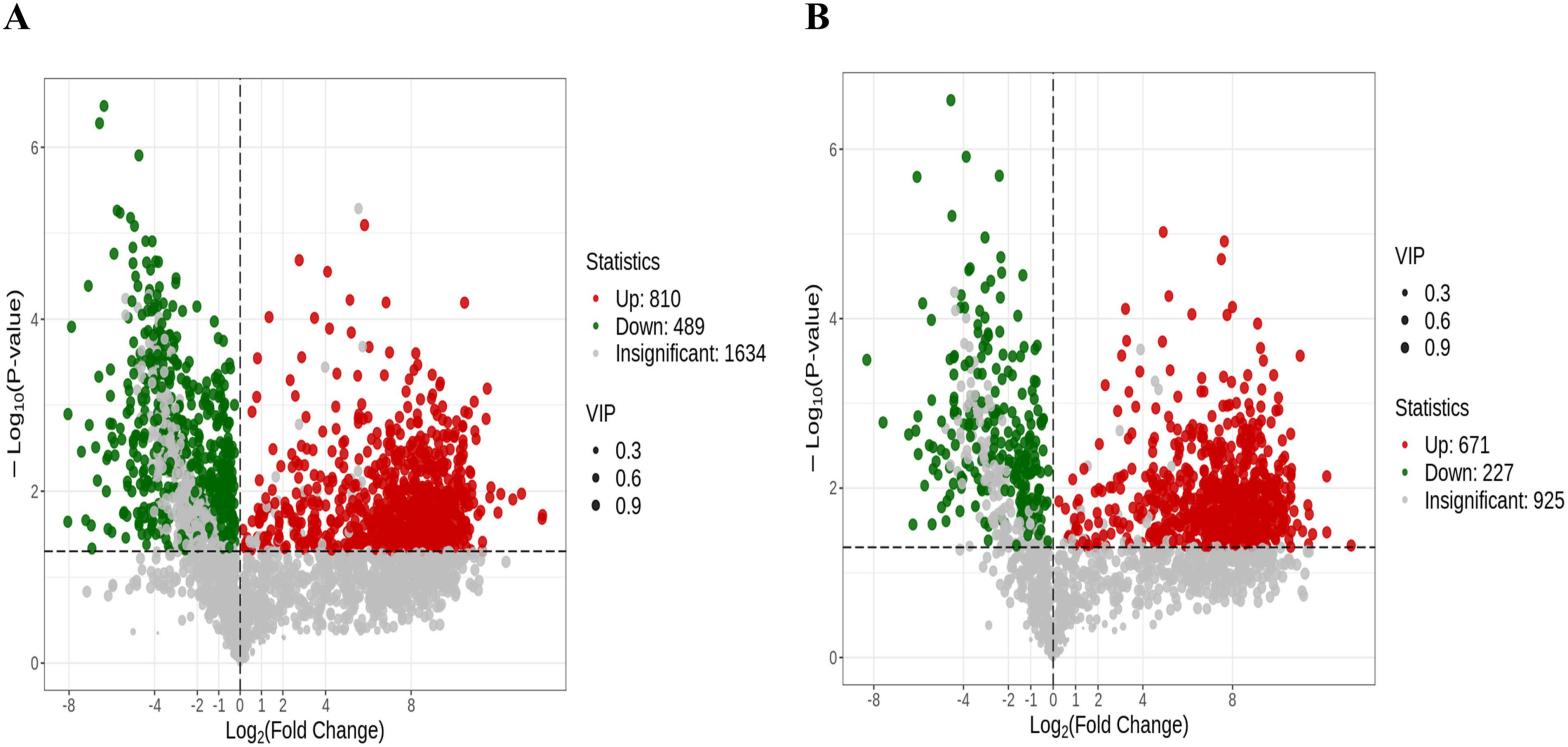
The differentially expressed metabolites in Volcano plot. The volcano plots depict all significantly altered metabolites (VIP > 1, *p* < 0.05) under the ESI+ **(A)** and ESI− **(B)**. Each dot in the volcano plots represents an individual metabolite: green dots denote downregulated differential metabolites, red dots represent upregulated differential metabolites, and gray dots represent detected metabolites with no significant differences. The *x*-axis indicates the logarithm of the fold change in the relative content of a specific metabolite between the two sample groups, while the y-axis represents the level of statistical significance.

In the OPLS-DA model, the VIP metric served to evaluate the contribution and explanatory power of each metabolite in distinguishing sample categories among different taxonomic groups, thereby facilitating the identification of biologically relevant differential metabolites. Significant differential metabolites were selected based on a VIP threshold greater than 1.0, combined with a statistical significance level of *p* < 0.05. A total of 2,197 metabolites were significantly altered upon PRV infection. The distribution of these metabolites was as follows: 810 increased and 489 decreased in the ESI+ mode (Figure 2A), and 671 increased and 227 decreased in the ES− mode (Figure 2B).

**Figure 3:**
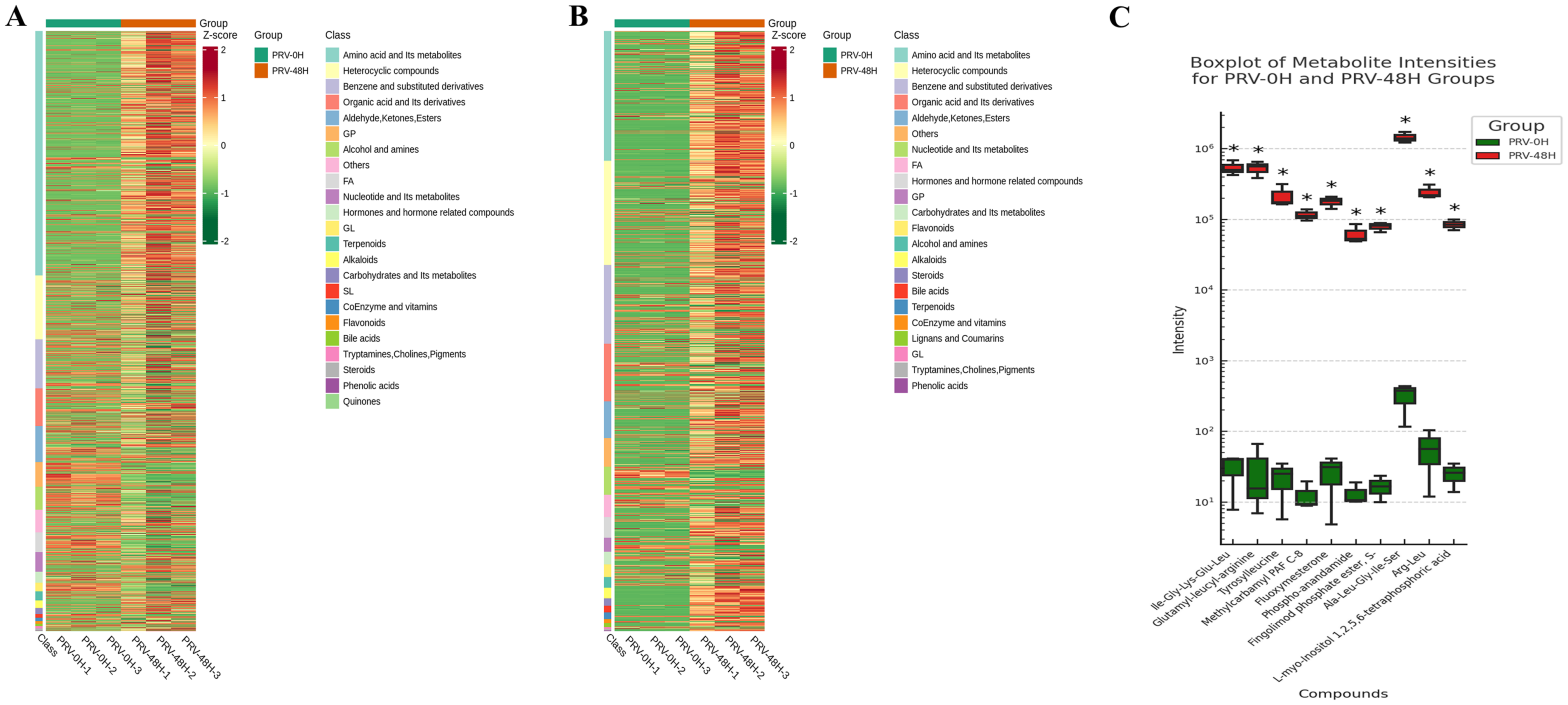
Analysis of differentially expressed metabolites. Heatmaps displaying the relative abundance (*Z*-score normalized) of all significantly altered metabolitesin ESI+ **(A)** and ESI− **(B)** modes. Rows represent metabolites, and columns represent biological replicates (*n* = 3 per group). **(C)** Box plots showing the normalized intensity distributions of 10 representative differentially abundant metabolites. Data are presented for each replicate in the PRV-0H and PRV-48H groups. Statistical significance was determined by Student’s *t*-test (*p < 0.05).

To dissect metabolic reprogramming during pseudorabies virus (PRV) infection, we performed hierarchical cluster analysis (HCA) on metabolite profiles of PK-15 cells at 0hpi (PRV-0H, control) and 48hpi (PRV-48H, infected). Heatmaps (Figures 3A,B) revealed distinct metabolic signatures between groups. In PRV-48H, metabolites clustered into characteristic patterns: amino acid and its metabolites, heterocyclic compounds showed consistent upregulation trends, while glycerophospholipids (GPs) were predominantly down-regulated, indicating PRV-driven metabolic skew. The boxplot of metabolite intensities (Figure 3C) visualized individual compound changes. Key metabolites like Ile-Gly-Lys-Glu-Leu, Glutamyl-leucine exhibited elevated intensities in PRV-48H, whereas L-myo-inositol 1,2,3,4,5-tetrakisphosphoric acid and related species showed differential abundance patterns, with statistically significant alterations (marked by *). These data demonstrate that PRV infection at 48hpi triggers profound metabolic reprogramming in host cells, remodeling metabolite classes and key compound abundances, which may support viral replication and pathogenesis.

### KEGG pathway enrichment analysis of differential metabolites

**Figure 4:**
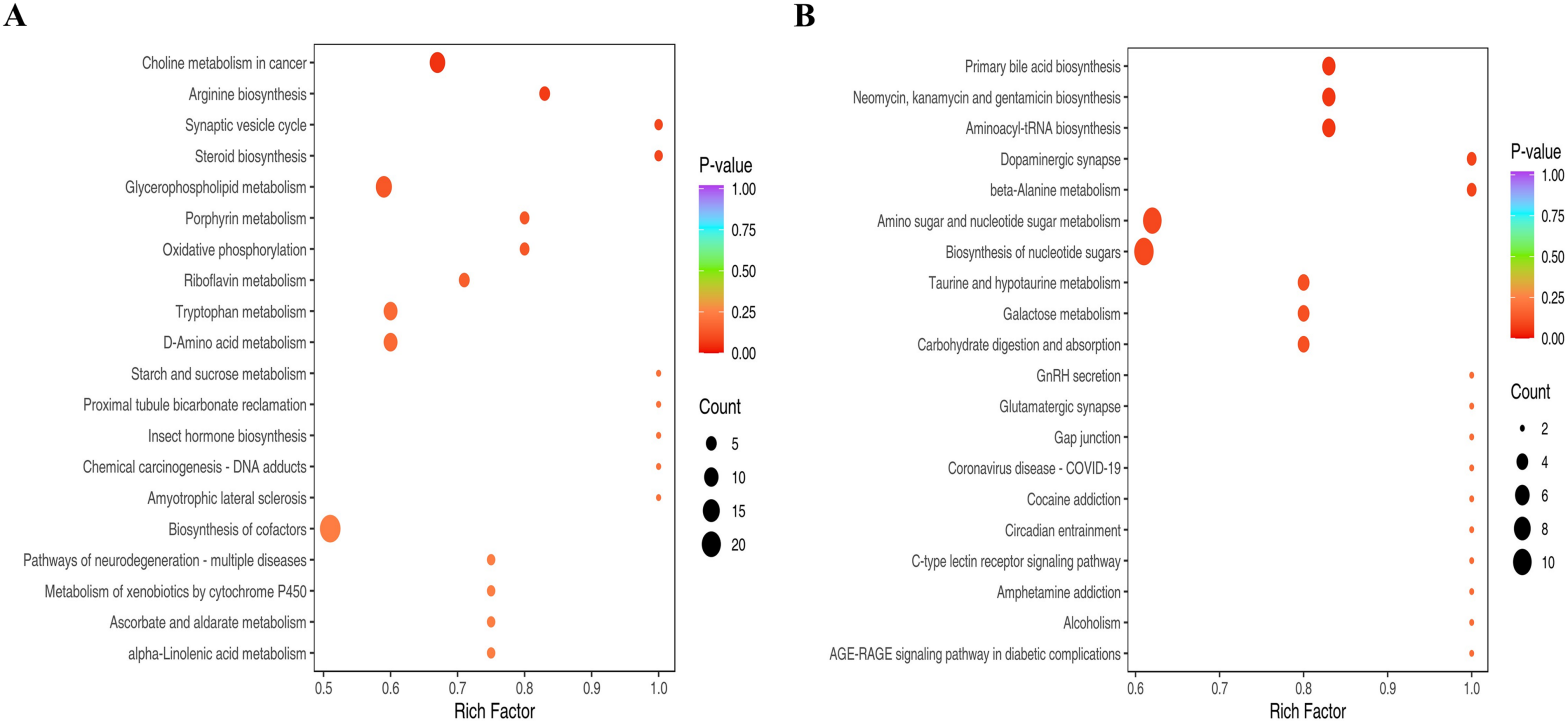
The KEGG analysis. **(A)** Pathways enriched for metabolites detected in ESI+. **(B)** Pathways enriched for metabolites detected in ESI−. The abscissa represents the Rich Factor corresponding to each pathway, and the ordinate is the pathway name (sorted by *p*-value). The color of the dots indicates the p-value, with redder dots representing more significant enrichment. The size of the dots represents the number of differential metabolites enriched.

To identify metabolic networks perturbed by PRV infection, we performed KEGG pathway enrichment analysis on differential metabolites from PRV-infected PK-15 cells at 48 hpi. The analysis was conducted separately for metabolites detected in positive (ESI+) and negative (ESI-) electrospray ionization modes, which can influence the detectability of different metabolite classes. This analysis revealed several pathways that were significantly enriched among the altered metabolites (Figures 4A,B).

Pathways significantly enriched among metabolites detected in ESI + mode included glycerophospholipid metabolism and arginine biosynthesis (Figure 4A). The enrichment of glycerophospholipid metabolism suggests a substantial perturbation in host membrane lipid dynamics. This is consistent with the extensive membrane biogenesis required for the replication of enveloped viruses like PRV, which undergoes secondary envelopment at intracellular membranes. The enrichment of arginine biosynthesis likely reflects an increased demand for this amino acid to support robust viral protein synthesis.

Among metabolites detected in ESI- mode, significantly enriched pathways included aminoacyl-tRNA biosynthesis, amino sugar and nucleotide sugar metabolism, and beta-alanine metabolism (Figure 4B). The enrichment of aminoacyl-tRNA and nucleotide sugar metabolism pathways indicates a heightened demand for translational capacity and protein glycosylation, essential processes for producing viral proteins and glycoproteins. The enrichment of the dopaminergic synapse pathway is noted; this KEGG term encompasses core metabolites involved in catecholamine synthesis and metabolism (e.g., dopamine, L-tyrosine). In the context of PK-15 kidney cells, this enrichment is interpreted as an alteration in the biochemistry of these shared small molecules, rather than as evidence of specific neuronal synapse disruption. Similarly, the enrichment of beta-alanine metabolism points to potential changes in coenzyme A and pantothenate metabolism, which are central to lipid and energy metabolism.

Collectively, the pathway enrichment analysis indicates that PRV infection induces a broad reprogramming of host metabolism, affecting pathways crucial for membrane biogenesis, protein synthesis, post-translational modification, and central carbon metabolism.

### Pronounced enrichment of nucleotide metabolism during PRV infection

**Figure 5:**
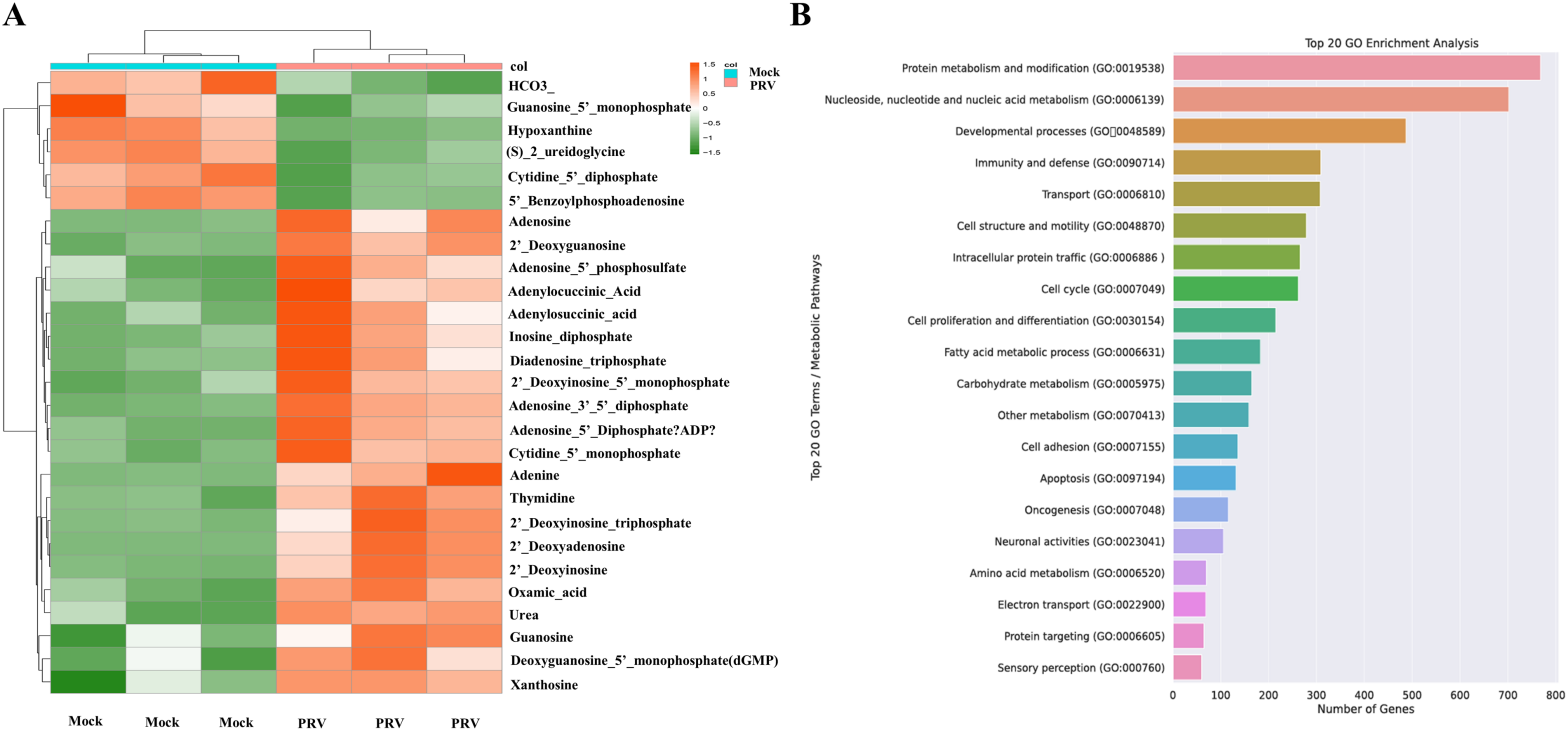
**(A)** The heatmap shows the changes of nucleotides. **(B)** GO enrichment analysis.

Nucleotides serve as crucial substrates for cellular metabolism and proliferation. Our study revealed a significant upregulation of various nucleotides 48 h post-infection, aligning with the viral replication process. This section delves into the specific nucleotides that were altered and their implications in the context of PRV infection. Among the nucleotides that exhibited a marked increase in levels were adenosine, guanosine, adenine, and xanthosine. These nucleotides are key components of nucleic acid, underscoring their importance in the viral replication machinery (Figure 5A). The observed upregulation of these nucleotides is consistent with the requirements of viral replication. Our findings suggested that PRV infection may activate nucleotide metabolism to facilitate its replication. To confirm our findings, we performed an enrichment analysis on PRV-infected transcriptomic data from the GSE8676 dataset. The results were in line with our metabolomic data, further validating the significant enrichment of nucleotide metabolism during PRV infection (Figure 5B). The pronounced enrichment of nucleotide metabolism pathways opens potential avenues for targeted antiviral therapies. Inhibitors of key enzymes involved in nucleotide synthesis could serve as effective antiviral agents by disrupting the viral replication process.

### Quantitative PCR validation of metabolomic data in the context of nucleotide metabolism during PRV infection

**Figure 6:**
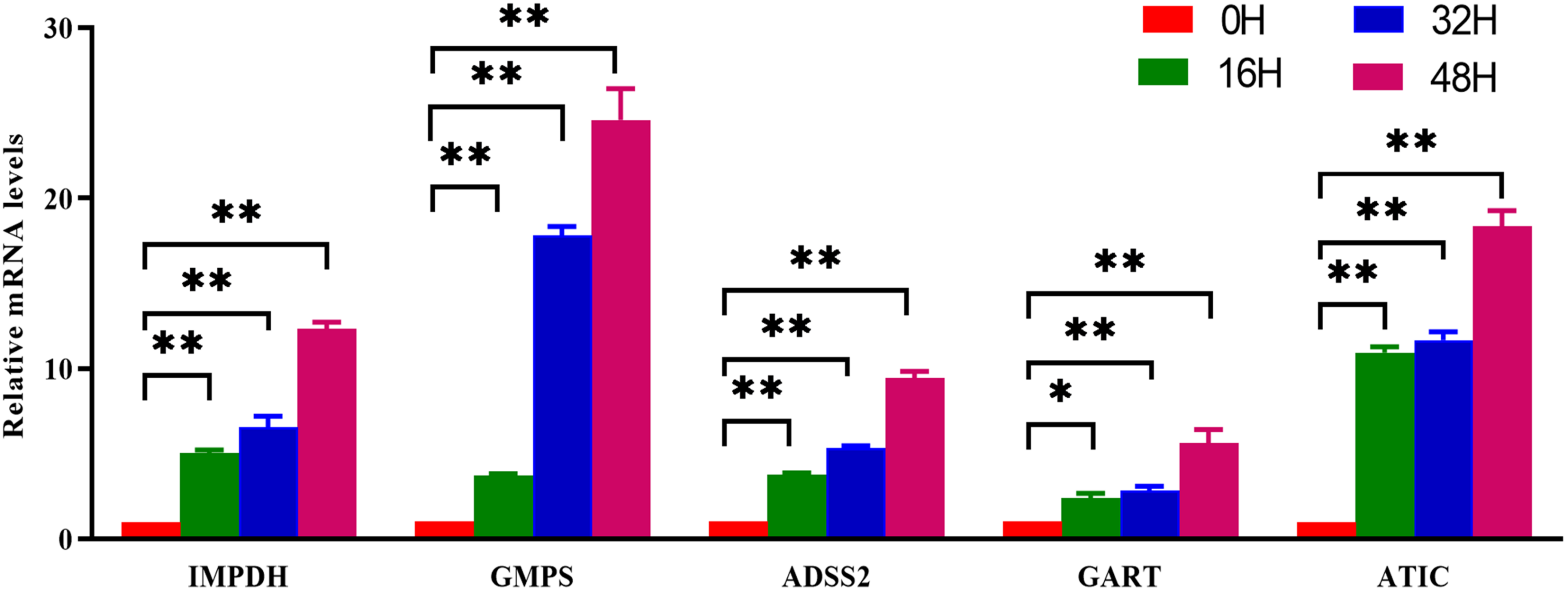
Validation of nucleotide metabolism related genes by qPCR. PK-15 cells were infected with PRV (RA strain, MOI = 1) or mock-infected and collected at the indicated time points. Total RNA was extracted and reverse-transcribed, followed by qRT-PCR analysis with gene-specific primers. Expression levels of purine biosynthesis genes (IMPDH, GMPS, ADSS2, GART, ATIC) were normalized to GAPDH and calculated using the 2^(–ΔΔCt) method relative to the 0 hpi control. Data are presented as mean ± S.D. from three independent biological replicates (*n* = 3). *p < 0.05, **p < 0.01.

To establish a direct link between the metabolomic upregulation of nucleotide metabolism and the transcriptional activation of key metabolic enzymes, we performed quantitative real-time PCR (qRT-PCR) to profile the mRNA levels of critical purine metabolism genes in PRV-infected cells over time. The genes analyzed—inosine monophosphate dehydrogenase (IMPDH), guanosine monophosphate synthase (GMPS), adenylosuccinate synthase 2 (ADSS2), phosphoribosylglycinamide formyltransferase (GART), and 5-aminoimidazole-4-carboxamide ribonucleotide formyltransferase (ATIC)—are central to *de novo* purine biosynthesis, a pathway strongly enriched in our metabolomic analysis. PRV infection induced robust, time-dependent upregulation of five key purine metabolism genes (Figure 6). At 0 hpi (control), all genes showed low baseline expression. By 16 hpi, IMPDH, GMPS, and ATIC were subtly but significantly upregulated (all *p* < 0.05), indicating early activation of purine biosynthesis to support initial viral replication. At 32 hpi, expression of these genes further escalated (all *p* < 0.01 for relevant comparisons), reflecting enhanced metabolic reprogramming as infection progresses. This mid-infection upregulation coincided with the metabolomic peak of nucleotides (e.g., adenosine, guanosine), highlighting coordinated metabolic and transcriptional responses. By 48 hpi, all genes reached maximal expression (*p* < 0.01). This confirmed PRV drives prolonged, intensified activation of purine metabolism genes, fueling de novo nucleotide biosynthesis crucial for peak viral DNA replication.

## Discussion

Viruses have evolved intricate strategies to hijack host metabolism, and understanding these interactions is pivotal for unraveling pathogenic mechanisms and identifying therapeutic targets (Chen et al., 2022; Yang et al., 2017). Our metabolomic analysis of PRV-infected PK-15 cells reveals a profound reprogramming of host metabolism, with nucleotide metabolism emerging as a central hub for viral replication. These findings not only deepen our understanding of PRV-host interactions but also highlight conserved and unique features of alphaherpesvirus metabolic manipulation (Frasson et al., 2019; Albano et al., 2025).

A defining observation of our study is the robust upregulation of nucleotide metabolism during PRV infection. At 48 hpi, key nucleotides (adenosine, guanosine) and their precursors (adenine, xanthosine) are significantly enriched, accompanied by time-dependent upregulation of critical enzymes in purine metabolism: IMPDH, GMPS, ADSS2, GART, and ATIC. This coordinated activation of metabolic intermediates and their synthetic enzymes underscores a deliberate strategy by PRV to enhance nucleotide availability, a prerequisite for viral DNA replication (Tang et al., 2023; Lindsey et al., 2021; Xu et al., 2024, 2022a,b).

This finding aligns with broader principles of herpesvirus biology: alphaherpesviruses, including herpes simplex virus (HSV) and human cytomegalovirus (HCMV), are known to upregulate nucleotide metabolism to support their lytic replication cycles (Bogani and Boehmer, 2008; Luganini et al., 2021; Tanaka et al., 1975; Munger et al., 2008). For example, HSV activates *de novo* pyrimidine synthesis by inducing the expression of dihydroorotate dehydrogenase (DHODH) (Luganini et al., 2021), whereas HCMV upregulates nucleotide biosynthesis through the ribose-5-phosphate pathway (Tanaka et al., 1975, Munger et al., 2008). Our work extends this paradigm to PRV, demonstrating that it leverages both metabolic and transcriptional layers to boost nucleotide pools. Notably, the substantial upregulation of purine metabolites in PRV-infected cells indicates that nucleotide metabolism is not merely a passive participant but rather a rate-limiting hub for PRV replication.

The reliance on de novo nucleotide synthesis (rather than salvage) is particularly striking. IMPDH, a rate-limiting enzyme in guanine nucleotide biosynthesis, is upregulated 12-fold at 48 hpi, mirroring observations in influenza A virus (IAV) infection where IMPDH inhibition abrogates viral replication (Hu et al., 2017). This conservation across diverse viruses highlights IMPDH and related enzymes as pan-antiviral targets. Our data raise the intriguing possibility that PRV encodes factors (e.g., viral transcription activators or host kinase modulators) that directly upregulate these metabolic enzymes, a hypothesis warranting future investigation into viral protein-metabolic enzyme interactions.

In contrast to nucleotide metabolism, GPs—key components of cellular membranes—were markedly downregulated in PRV-infected cells. GPs, including phosphatidylethanolamine and phosphatidylinositol, are critical for membrane integrity, organelle function, and signaling (Dawaliby et al., 2016; Chen et al., 2025). We hypothesize that this depletion may serve dual purposes. First, the reduction in host membrane lipids could facilitate the extensive membrane remodeling required for processes such as viral secondary envelopment at intracellular compartments, a prerequisite for mature virion formation whose primary egress route is via the constitutive secretory pathway (Liu et al., 2020). Second, and more speculatively, the downregulation of GP precursors might indirectly influence host innate immunity. This hypothesis is grounded in literature indicating that certain GP-derived signaling molecules, notably phosphatidic acid, can serve as platforms for activating antiviral pathways, including IFN-*β* induction (Jin et al., 2023). A global reduction in their precursor pools could therefore potentially dampen such responses, a strategy analogous to that employed by other viruses like PEDV (Corn et al., 2020; Shi et al., 2024). It is crucial to note, however, that this proposed link to immune evasion remains a testable hypothesis, as our current metabolomic data do not directly measure the flux through these specific signaling pathways. Notably, the downregulation of GPs in PRV infection presents a contrast to some coronavirus infections, where specific viral proteins can enhance glycerophospholipid synthesis to support replication (Yue et al., 2023), suggesting distinct viral lipid manipulation strategies. Future studies employing targeted lipidomics and signaling assays are warranted to determine whether PRV actively degrades GPs, redirects lipid precursors, and functionally impairs lipid-mediated innate immune signaling.

Our findings indicate that PRV infection creates a specific vulnerability in the host’s nucleotide metabolism, as evidenced by the pronounced upregulation of key *de novo* purine biosynthesis enzymes, IMPDH and GMPS. This suggests that PRV replication is critically dependent on an enhanced flux through this pathway. To functionally validate this dependency, future studies should employ specific IMPDH inhibitors—such as mycophenolic acid or merimepodib. The antiviral effect of these inhibitors, and its specific reversal by exogenous guanosine supplementation in rescue experiments, would provide direct causal evidence linking this metabolic pathway to viral replication. Targeting host nucleotide metabolism offers a potential strategic advantage by potentially reducing the risk of viral resistance, a common issue with therapies directed against viral proteins. However, the critical challenge lies in achieving selectivity, as proliferating host cells also require nucleotides. The therapeutic window may arise from PRV’s heightened reliance on de novo synthesis, potentially sparing the salvage pathways utilized by normal cells. Combining metabolomic profiling with high-throughput drug screening in infected cells could therefore accelerate the identification of inhibitors that selectively target these virus-induced metabolic fluxes.

This study has several limitations that should be considered. The metabolomic analysis was conducted with a limited sample size (*n* = 3) in a single, non-neuronal cell line (PK-15), which may not fully represent the metabolic landscape in the primary neuronal targets of PRV. Our detailed analysis focused on the peak of viral replication (48 hpi) to capture the most pronounced metabolic state, although we note that earlier timepoints (24, 36 hpi) were also profiled and will be the subject of future work on the dynamics of early metabolic reprogramming. The potential contribution of late-stage cytopathic effects at this timepoint, combined with the lack of direct correlation with viral titers, metabolic flux assays, dNTP pool measurements, or targeted lipidomics, means that the functional consequences of the observed metabolic shifts require further validation. Future studies employing time-course analyses in primary neuronal cells or animal models will be essential to delineate the core virus-induced metabolic program from secondary effects and to confirm the generalizability of these findings across different PRV strains.

In summary, our work uncovers a central role for nucleotide metabolism in PRV replication, positioning it as a key mediator of virus-host metabolic crosstalk. By highlighting conserved and unique features of PRV-induced metabolic reprogramming, these findings advance our understanding of alphaherpesvirus pathogenesis and provide a foundation for developing metabolism-targeted antivirals.

## Data Availability

The raw data supporting the conclusions of this article will be made available by the authors, without undue reservation.

## Footnotes

^1^ www.r-project.org

^2^ http://www.kegg.jp/kegg/compound/

^3^ http://www.kegg.jp/kegg/pathway.html

## References

[ref1] Albano C. Trifirò L. Hewelt-Belka W. Cairns D. M. Pasquero S. Griffante G. . (2025). The impact of fatty acid synthase on HSV-1 infection dynamics. PLoS Pathog. 21:e1013068. doi: 10.1371/journal.ppat.1013068, PMID: 40327680 PMC12084038

[ref2] Bai Y. Li L. Shan T. Zhang Y. Chen X. Gao F. . (2020). Proteasomal degradation of nonstructural protein 12 by RNF114 suppresses porcine reproductive and respiratory syndrome virus replication. Vet. Microbiol. 246:108746. doi: 10.1016/j.vetmic.2020.108746, PMID: 32605740

[ref3] Bauermeister A. Mannochio-Russo H. Costa-Lotufo L. V. Jarmusch A. K. Dorrestein P. C. (2022). Mass spectrometry-based metabolomics in microbiome investigations. Nat. Rev. Microbiol. 20, 143–160. doi: 10.1038/s41579-021-00621-9, PMID: 34552265 PMC9578303

[ref4] Beale D. J. Pinu F. R. Kouremenos K. A. Poojary M. M. Narayana V. K. Boughton B. A. . (2018). Review of recent developments in GC-MS approaches to metabolomics-based research. Metabolomics 14:152. doi: 10.1007/s11306-018-1449-2, PMID: 30830421

[ref5] Bogani F. Boehmer P. E. (2008). The replicative DNA polymerase of herpes simplex virus 1 exhibits apurinic/apyrimidinic and 5′-deoxyribose phosphate lyase activities. Proc. Natl. Acad. Sci. USA 105, 11709–11714. doi: 10.1073/pnas.0806375105, PMID: 18695225 PMC2575316

[ref6] Chen X. He R. Xiong H. Wang R. Yin Y. Chen Y. . (2025). Quantitative profiling of lipid transport between organelles enabled by subcellular photocatalytic labelling. Nat. Chem. 17, 1534–1545. doi: 10.1038/s41557-025-01886-w, PMID: 40770077

[ref7] Chen X. Kong N. Xu J. Wang J. Zhang M. Ruan K. . (2021). Pseudorabies virus UL24 antagonizes OASL-mediated antiviral effect. Virus Res. 295:198276. doi: 10.1016/j.virusres.2020.198276, PMID: 33476694

[ref8] Chen X. Shan T. Sun D. Zhai H. Dong S. Kong N. . (2022). Host zinc-finger CCHC-type containing protein 3 inhibits pseudorabies virus proliferation by regulating type I interferon signaling. Gene 827:146480. doi: 10.1016/j.gene.2022.146480, PMID: 35390445

[ref9] Chen X. Wang S. Wu M. Zhao Y. (2023). Role of succinylation in pseudorabies virus infection. J. Virol. 97:e0179022. doi: 10.1128/jvi.01790-22, PMID: 36975827 PMC10134788

[ref10] Corn K. C. Windham M. A. Rafat M. (2020). Lipids in the tumor microenvironment: from cancer progression to treatment. Prog. Lipid Res. 80:101055. doi: 10.1016/j.plipres.2020.101055, PMID: 32791170 PMC7674189

[ref11] Dawaliby R. Trubbia C. Delporte C. Noyon C. Ruysschaert J. M. VAN Antwerpen P. . (2016). Phosphatidylethanolamine is a key regulator of membrane fluidity in eukaryotic cells. J. Biol. Chem. 291, 3658–3667. doi: 10.1074/jbc.M115.706523, PMID: 26663081 PMC4751403

[ref12] Frasson I. Nadai M. Richter S. N. (2019). Conserved G-quadruplexes regulate the immediate early promoters of human alphaherpesviruses. Molecules 24:2375. doi: 10.3390/molecules24132375, PMID: 31252527 PMC6651000

[ref13] Gong Y. Tang N. Liu P. Sun Y. Lu S. Liu W. . (2022). Newcastle disease virus degrades SIRT3 via PINK1-PRKN-dependent mitophagy to reprogram energy metabolism in infected cells. Autophagy 18, 1503–1521. doi: 10.1080/15548627.2021.1990515, PMID: 34720029 PMC9298456

[ref14] Hu J. Ma L. Wang H. Yan H. Zhang D. Li Z. . (2017). A novel benzo-heterocyclic amine derivative N30 inhibits influenza virus replication by depression of Inosine-5'-Monophospate dehydrogenase activity. Virol. J. 14:55. doi: 10.1186/s12985-017-0724-6, PMID: 28298229 PMC5353780

[ref15] Jin X. Xia T. Luo S. Zhang Y. Xia Y. Yin H. (2023). Exosomal lipid PI4P regulates small extracellular vesicle secretion by modulating intraluminal vesicle formation. J. Extracell. Vesicles 12:e12319. doi: 10.1002/jev2.12319, PMID: 37021404 PMC10076970

[ref16] Johnson C. H. Ivanisevic J. Siuzdak G. (2016). Metabolomics: beyond biomarkers and towards mechanisms. Nat. Rev. Mol. Cell Biol. 17, 451–459. doi: 10.1038/nrm.2016.25, PMID: 26979502 PMC5729912

[ref17] Lindsey A. R. I. Bhattacharya T. Hardy R. W. Newton I. L. G. (2021). Wolbachia and virus alter the host transcriptome at the interface of nucleotide metabolism pathways. MBio 12:e03472-20. doi: 10.1128/mBio.03472-20, PMID: 33563832 PMC7885120

[ref18] Liu Y. T. Shivakoti S. Jia F. Tao C. L. Zhang B. Xu F. . (2020). Biphasic exocytosis of herpesvirus from hippocampal neurons and mechanistic implication to membrane fusion. Cell Discov. 6:2. doi: 10.1038/s41421-019-0134-6, PMID: 31969988 PMC6957672

[ref19] Luganini A. Sibille G. Mognetti B. Sainas S. Pippione A. C. Giorgis M. . (2021). Effective deploying of a novel DHODH inhibitor against herpes simplex type 1 and type 2 replication. Antivir. Res. 189:105057. doi: 10.1016/j.antiviral.2021.105057, PMID: 33716051

[ref20] Mao J. Huang X. Shan Y. Xu J. Gao Q. Xu X. . (2022). Transcriptome analysis revealed inhibition of lipid metabolism in 2-D porcine enteroids by infection with porcine epidemic diarrhea virus. Vet. Microbiol. 273:109525. doi: 10.1016/j.vetmic.2022.109525, PMID: 35963027

[ref21] Munger J. Bennett B. D. Parikh A. Feng X. J. Mcardle J. Rabitz H. A. . (2008). Systems-level metabolic flux profiling identifies fatty acid synthesis as a target for antiviral therapy. Nat. Biotechnol. 26, 1179–1186. doi: 10.1038/nbt.1500, PMID: 18820684 PMC2825756

[ref22] Pang Y. Li C. Wang Y. Liu J. Su G. Duan C. . (2023). Porcine reproductive and respiratory syndrome virus infection manipulates central carbon metabolism. Vet. Microbiol. 279:109674. doi: 10.1016/j.vetmic.2023.109674, PMID: 36739813

[ref23] Pei G. Chen L. Zhang W. (2017). WGCNA application to proteomic and Metabolomic data analysis. Methods Enzymol. 585, 135–158. doi: 10.1016/bs.mie.2016.09.016, PMID: 28109426

[ref24] Shi X. Zhang Q. Yang N. Wang Q. Zhang Y. Xu X. (2024). PEDV inhibits HNRNPA3 expression by miR-218-5p to enhance cellular lipid accumulation and promote viral replication. MBio 15:e0319723. doi: 10.1128/mbio.03197-23 38259103 PMC10865979

[ref25] Sun L. Tang Y. Yan K. Yu J. Zou Y. Xu W. . (2019). Differences in neurotropism and neurotoxicity among retrograde viral tracers. Mol. Neurodegener. 14:8. doi: 10.1186/s13024-019-0308-6, PMID: 30736827 PMC6368820

[ref26] Tan L. Yao J. Yang Y. Luo W. Yuan X. Yang L. . (2021). Current status and challenge of pseudorabies virus infection in China. Virol. Sin. 36, 588–607. doi: 10.1007/s12250-020-00340-0, PMID: 33616892 PMC7897889

[ref27] Tanaka S. Furukawa T. Plotkin S. A. (1975). Human cytomegalovirus stimulates host cell RNA synthesis. J. Virol. 15, 297–304. doi: 10.1128/jvi.15.2.297-304.1975, PMID: 163357 PMC354453

[ref28] Tang N. Chen P. Zhao C. Liu P. Tan L. Song C. . (2023). Newcastle disease virus manipulates mitochondrial MTHFD2-mediated nucleotide metabolism for virus replication. J. Virol. 97:e0001623. doi: 10.1128/jvi.00016-23, PMID: 36794935 PMC10062132

[ref29] Xu L. Chen Z. Zhang Y. Cui L. Liu Z. Li X. . (2022a). P53 maintains gallid alpha herpesvirus 1 replication by direct regulation of nucleotide metabolism and ATP synthesis through its target genes. Front. Microbiol. 13:1044141. doi: 10.3389/fmicb.2022.1044141, PMID: 36504811 PMC9729838

[ref30] Xu L. Wang Z. Chen Z. Cui L. Liu Z. Liang Y. . (2022b). PFT-α inhibits gallid alpha herpesvirus 1 replication by repressing host nucleotide metabolism and ATP synthesis. Vet. Microbiol. 269:109435. doi: 10.1016/j.vetmic.2022.109435, PMID: 35462119

[ref31] Xu Y. Yi H. Kuang Q. Zheng X. Xu D. Gong L. . (2024). Nucleotide metabolism-related host proteins RNA polymerase II subunit and uridine phosphorylase 1 interacting with porcine epidemic diarrhea virus N proteins affect viral replication. Front. Vet. Sci. 11:1417348. doi: 10.3389/fvets.2024.1417348, PMID: 38933700 PMC11200923

[ref32] Yang S. Pei Y. Zhao A. (2017). iTRAQ-based proteomic analysis of porcine kidney epithelial PK15 cells infected with pseudorabies virus. Sci. Rep. 7:45922. doi: 10.1038/srep45922, PMID: 28374783 PMC5379687

[ref33] Yu Z. Q. Tong W. Zheng H. Li L. W. Li G. X. Gao F. . (2017). Variations in glycoprotein B contribute to immunogenic difference between PRV variant JS-2012 and Bartha-K61. Vet. Microbiol. 208, 97–105. doi: 10.1016/j.vetmic.2017.07.019, PMID: 28888658

[ref34] Yue M. Hu B. Li J. Chen R. Yuan Z. Xiao H. . (2023). Coronaviral ORF6 protein mediates inter-organelle contacts and modulates host cell lipid flux for virus production. EMBO J. 42:e112542. doi: 10.15252/embj.2022112542, PMID: 37218505 PMC10308351

